# Can Botulinum Toxin-A Contribute to Reconstructing the Physiological Homeostasis of the Masticatory Complex in Short-Faced Patients during Occlusal Therapy? A Prospective Pilot Study

**DOI:** 10.3390/toxins14060374

**Published:** 2022-05-28

**Authors:** Xin Li, Xiaoyan Feng, Juan Li, Xinyu Bao, Jinghong Xu, Jun Lin

**Affiliations:** 1 Department of Stomatology, The First Affiliated Hospital, College of Medicine, Zhejiang University, Hangzhou 310003, China; 22018628@zju.edu.cn (X.L.); 21718013@zju.edu.cn (X.F.); lijuan5650@zju.edu.cn (J.L.); 22118751@zju.edu.cn (X.B.); 2 Department of Plastic Surgery, The First Affiliated Hospital, College of Medicine, Zhejiang University, Hangzhou 310003, China; 1304017@zju.edu.cn

**Keywords:** botulinum toxin-A, masticatory complex, cone-beam computed tomography, masseter muscle, short-faced patients

## Abstract

The physiological homeostasis of the masticatory complex in short-faced patients is too robust to be disintegrated and reconstructed due to the powerful masseter muscle. This study innovatively introduced the botulinum toxin-A (BTX-A) into the field of dental occlusal treatment, providing a novel and minimally invasive therapy perspective for the two major clinical problems in these patients (low treatment efficiency and high rates of complications). In total, 10 adult patients with skeletal low angle seeking occlusal treatment (age: 27.0 ± 6.1 years; 4 males and 6 females) were administered 30–50 U of BTX-A in each masseter muscle and evaluated before and 3 months after injection based on cone-beam computed tomography (CBCT). We found a significant reduction in the thickness of the masseter muscle (MMT) (*p* < 0.0001). With regards to occlusion, we found a significant increase in the height of the maxillary second molar (U7-PP) (*p* < 0.05) with significantly flattened occlusal curves (the curve of Spee [COS] (*p* < 0.01), and the curve of Wilson [COW] (*p* < 0.05)). Furthermore, the variations in the temporomandibular joint exhibited a significant reduction in the anterior joint space (AJS) (*p* < 0.05) and superior joint space (SJS) (*p* < 0.05). In addition, the correlation analysis of the masticatory complex provided the basis for the following multiple regression equation: MMT = 10.08 − 0.11 COW + 2.73 AJS. The findings from our pilot study indicate that BTX-A, as a new adjuvant treatment attempt of occlusal therapy for short-faced patients, can provide a more favorable muscular environment for subsequent occlusal therapy through the adjustment of the biting force and may contribute to the reconstruction of healthier homeostasis of the masticatory complex. However, further research is required to establish the reliability and validity of these findings.

## 1. Introduction

Since the birth of dentistry, the discussion on the target of occlusal treatment has been continuously progressing [1,2,3]. In the early stage, dentists only focused on the teeth and occlusal relationship, which was generally static and non-functional [4,5,6]. Until the prevalence of functional occlusion in recent years [7,8], the concept of complete dentistry was put forward [9], with the studies on dynamic occlusion and masticatory function valued by interdisciplinary oral researchers [1,2,3,10,11,12,13,14,15,16,17,18], especially in the fields of orthodontics [1,2,3], occlusal reconstruction [10,11], dental implant [12,13], periodontal disease [14,15], dental caries treatment [16], temporomandibular joint disorders (TMD) [17], bruxism [18], etc. Dentists have begun to realize the integrality of the masticatory system (MS) (i.e., the stomatognathic system (SS)), which is generally composed of teeth and occlusion, the temporomandibular joint (TMJ), neuromuscular factors, jaw, periodontal tissue, etc. [19]. Therefore, occlusal therapy is regarded as the process of dynamic reconstruction of each component, whose ultimate goal is to maintain the health and stability of the whole masticatory system [1,2,3,9,19].

**Figure 1:**
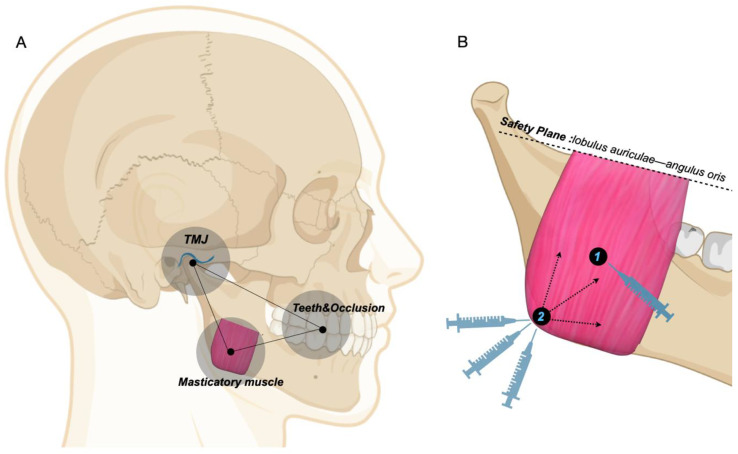
Schematic diagram of the masticatory complex and BTX-A injection method. (**A**) It demonstrates the triangle homeostasis of teeth and occlusion, temporomandibular joint (TMJ), and masticatory muscle. The three interrelate and have mutual influence under normal physiological conditions to guarantee the normal operation of the stomatognathic system, with any alteration beyond the body’s adaptability in one of the links resulting in occlusal diseases. (**B**) Each patient was administrated 30–50 U of BTX-A (Botox^®^, 100 U/vial, Allergan, Irvine, CA, USA) in each masseter muscle (30 G, 25 mm needle length), with a maximum bilateral difference of 5 U. Injection should be conducted below the safety plane connecting the lobulus auriculae and the angulus oris. Palpation was used to select the most significant location of masseter muscle swelling as the first point for injection, with the dose controlled at 15–30 U. The second point was the mandibular angle region, with injection upward from the mandibular edge in a fan-shaped manner. In general, 3 channels with less than 10 U each can cover the entire injection area.

The significant role of muscle factors in dental and maxillofacial development has been expounded by a lot of scholars [20,21]. It is proved that the occlusion and jaw are the consequence of the balance among multiple functional systems involving the masticatory system, respiratory system, etc., which also serves as the theoretical basis to prevent and correct jaw deformity and malocclusion in adolescence [22]. In addition, the function of TMJ is also closely related to the masticatory muscles, whose long-term discoordination and hyperactivity results in structural variation of TMJ [23], which was observed even in adult patients with a stable tendency [24]. Therefore, we conducted this study on the masticatory complex composed of the three (muscle-occlusion-joint complex) (Figure 1A). 

The masseter muscle is the most powerful masticatory muscle in the maxillofacial region, which ensures the stomatognathic system is fully stressed [21,22,25]. Specifically, variation in the masseter muscle can tremendously affect the function of the masticatory system, where weakness in the masseter muscle elicits the mandible to rotate clockwise, resulting in the opening of the mandible angle and the formation of high angle with a long-faced pattern [26]. In contrast, in short-faced patients with skeletal low angle, the masseter muscle is powerful and thick, which causes a square-shaped jaw accompanied by deep overbite and reduced vertical height [25,26,27,28,29]. As an extremely strong muscle-occlusion-joint triangular balance is too robust to be broken and reconstructed, it is hard for short-faced people to reestablish a new physiological homeostasis of the masticatory complex, indicating that occlusal treatment for these patients is awfully tough [29,30]. For example, it is generally rather difficult to increase the vertical height or open bite in occlusal therapy due to the powerful muscle-occlusion balance, and the curative effect is unstable with a high recurrence rate [31,32]. Moreover, these patients usually have a stout jaw and high bone density [21], which poses a challenge for successful tooth movement in orthodontic treatment, especially in cases of tooth extraction. In addition, because of the frequent impact of locking occlusion, posteriorly positioned mandible and condyle [33,34], steep occlusal curve and occlusal plane [35,36], patients are prone to posterior occlusion interference, which easily elicits bruxism or tooth wear, facial pain, muscle dysfunction, and TMD [17,18].

Therefore, the establishment of coordinated muscle function is the crux to effectively modify the occlusal relationship with long-term stability [10,37]. For dentists, two major technical difficulties impede the establishment of harmonious muscle function. One is the limitation of conventional dental treatment, for example, despite the multiple intervention measures with good clinical achievements for the occlusion, jaw, and TMJ, dentists seem to be powerless to adjust the neuromuscular factors. Although the occlusal splints can relax masticatory muscles while treating TMD [38,39], it is achieved indirectly by isolating the occlusal contact. Therefore, a minimally invasive, simple, and effective approach to directly intervene in neuromuscular factors is required, and botulinum toxin-A (BTX-A) can meet these treatment needs. BTX-A works by inhibiting the release of acetylcholine at the nerve–muscle junction [40] and has been applied in the auxiliary treatment of some diseases in the oral and maxillofacial region [41]. At present, its effectiveness in the treatment of bruxism [42,43], facial paralysis [44], TMD [45,46], neuropathic pain [42], sialorrhea [47], orofacial dystonia [48], and other diseases has been verified by high-level evidence-based medical evidence, providing a new direction and method for alternative treatment of oral diseases. However, the studies above [41,42,43,44,45,46,47,48] mostly focused on the field of oral and maxillofacial surgery, with insufficient attention given to occlusal therapy. Currently, only some animal experiments have been carried out [49,50,51,52,53] but almost no clinical studies.

Another issue is the difficulty in obtaining muscle diagnosis using dental imaging. Traditional two-dimensional dental imaging data can only present the hard tissue components with the possibility of “distortion” due to the influence of overlap and magnification. With the application of digitalization, cone-beam computed tomography (CBCT) and its three-dimensional reconstruction technology [54], which can reproduce both the soft and hard tissue in equal proportions [55,56,57], have been widely utilized in dental clinical work. CBCT can greatly meet the requirements of convenient diagnosis and treatment in dental clinics, with a much lower price, cost of time, and radiation, overcoming the disadvantages of traditional 3D imaging approaches such as CT [39,58] and MRI [59,60].

Based on CBCT images, this study preliminarily explored the feasibility of clinical application of BTX-A to reconstruct the physiological homeostasis of the masticatory complex in short-faced patients by researching the variation in the masticatory complex, aiming to provide an innovative and minimally invasive treatment perspective for the two major clinical problems, involving the low treatment efficiency (e.g., slow tooth movement, difficult bite opening, easy recurrence, etc.) and various potential complications (e.g., TMD, myofascial pain, bruxism, periodontal problems, etc.). In addition, the coordination mechanisms of the muscle-occlusion-joint complex are also discussed here by providing the regression equation for the first time, extending clinicians’ cognition of dynamic reconstruction of the stomatognathic system in occlusal therapy.

## 2. Patients and Methods

### 2.1. Subjects

Patients requiring orthodontic treatment in the Department of Stomatology, The First Affiliated Hospital of Zhejiang University Medical College from May 2021 to January 2022 were selected. In total, 10 eligible patients with an average age of 27.0 ± 6.1 years (4 males and 6 females) were selected according to the following criteria. Inclusion and exclusion criteria: (1) Short-faced patients with skeletal low angle (FMA< 24°) and benign masseter hypertrophy; (2) healthy adults, aged 18–35 years; (3) eruption of all permanent teeth (except the third molar); (4) a basically symmetrical maxillofacial region without significant deviation; (5) no malocclusion such as open bite or crossbite, and no serious dental and periodontal diseases; (6) no previous history record of maxillofacial surgery (such as orthognathic surgery, plastic surgery, etc.), orthodontic treatment, or prosthetic treatment; and (7) no masticatory system dysfunction, temporomandibular joint disorder syndrome, etc. This study was approved by the Clinical Research Ethics Committee of the First Affiliated Hospital, College of Medicine, Zhejiang University (protocol code: (2021) IIT (171) and date of approval: 10 March 2021). Before the study, all subjects were informed of the purpose of this study and gave informed consent.

### 2.2. BTX-A Injection Method

Each patient was treated with a single injection of BTX-A (Botox^®^, 100 U/vial, Allergan, Irvine, CA, USA), with 2 mL of NS added, diluting the concentration to 5 U/0.1mL. The patient was placed in the supine position and injected with 30–50 U/side into the masseter muscle (30 G, 25 mm needle length), with a maximum bilateral difference of 5 U. An expert plastic surgeon determined the specific injection dose of BTX-A based on his clinical expertise, and the injection was administered below the safety plane linking the lobulus auriculae and the angulus oris. Palpation was used to select the most significant location of masseter muscle swelling as the first point for injection, with the dose controlled at 15–30 U. The mandibular angle region was selected as the second point, with injection performed upward from the mandibular edge in a fan-shaped manner. In general, three channels with less than 10 U each can cover the entire injection area (Figure 1B). When injecting BTX-A, adrenaline (1:1000) should be prepared, followed by routine nursing care and short-term observation.

### 2.3. CBCT Measurement Items and Methods

**Figure 2:**
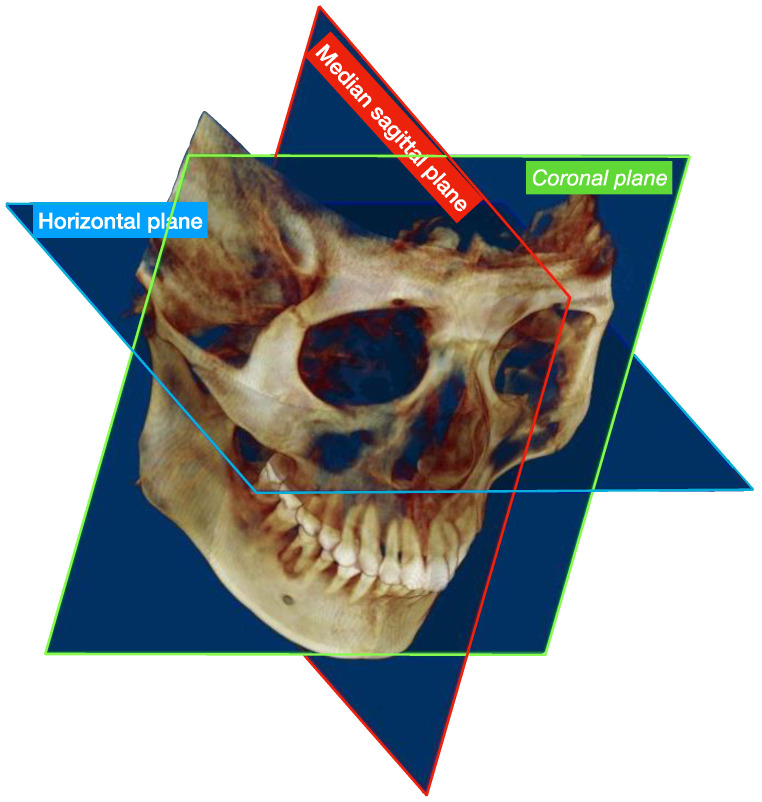
Schematic diagram of 3D reconstruction of CBCT. It displays the outcomes of the 3D reconstruction images using Dolphin Imaging 11.95 software (Chatsworth, Los Angeles, CA, USA) along with three calibration planes, which can be slid or rotated to obtain subsequent measurement planes for the measurement of various indexes.

CBCT (NewTom VGi, Verona, Italy) was taken in all patients 1 week before BTX-A injection and 3 months after injection. During the shooting, the patient was required to maintain the maximum intercuspal position, with consistent scanning parameters (tube voltage 110 kV, tube current 3.5 mA, exposure time 3.6 s, and definition 0.3 mm). The scanning ranged from the superior orbital edge to the lower mandibular edge. All CBCT data were saved in DICOM format and imported into Dolphin Imaging 11.95 software (Chatsworth, Los Angeles, CA, USA) for 3D reconstruction and analysis (Figure 2). In this study, the three components of the masticatory complex were measured and analyzed, and the data were measured twice by the same staff with an interval of one week, and the average value was taken. If the data difference between the two measurements exceeded 0.5 mm, one more measurement was carried out again.

#### 2.3.1. Masseter Muscle Index

**Figure 3:**
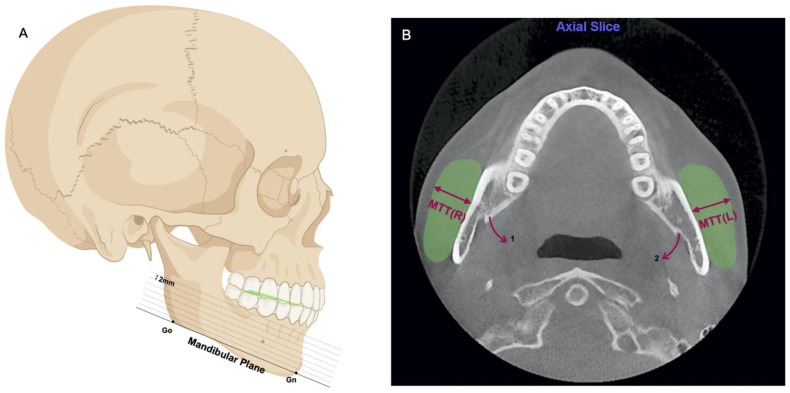
Measurement of the masseter muscle thickness (MTT). (**A**) The head position in the 3D reconstruction was required to be repositioned, ensuring the CBCT image was divided parallel to the mandibular plane (MP). The distance between each slice was 2mm; (**B**) The MMT of both sides was measured on the axial slice passing through the mandibular lingual structure (arrow 1), which is a sharp and thin bony piece in front of the mandibular foramen (arrow 2).

When measuring the masseter muscle thickness (MMT), the head position in the 3D reconstruction was required to be repositioned in three dimensions using line calibration, and the CBCT image was divided into several 2 mm slices parallel to the mandibular plane (MP) (Figure 3A). The MP was defined as the plane from the Gnathion point (Gn) to the Gonion point (Go). The MMT of both sides was measured on the axial slice passing through the mandibular lingual structure (a sharp and thin bony piece in front of the mandibular foramen) (Figure 3B), as previous studies [61] have suggested that the maximum section of the masseter muscles of most individuals is generally at the level of the mandibular foramen.

#### 2.3.2. Dental and Occlusal Indexes

There were 14 items in total, and the indexes in this part were divided into static occlusal indexes (6 posterior tooth height items, 4 transverse width items) and dynamic functional indexes (2 occlusal plane items and 2 occlusal curve items).

**Figure 4:**
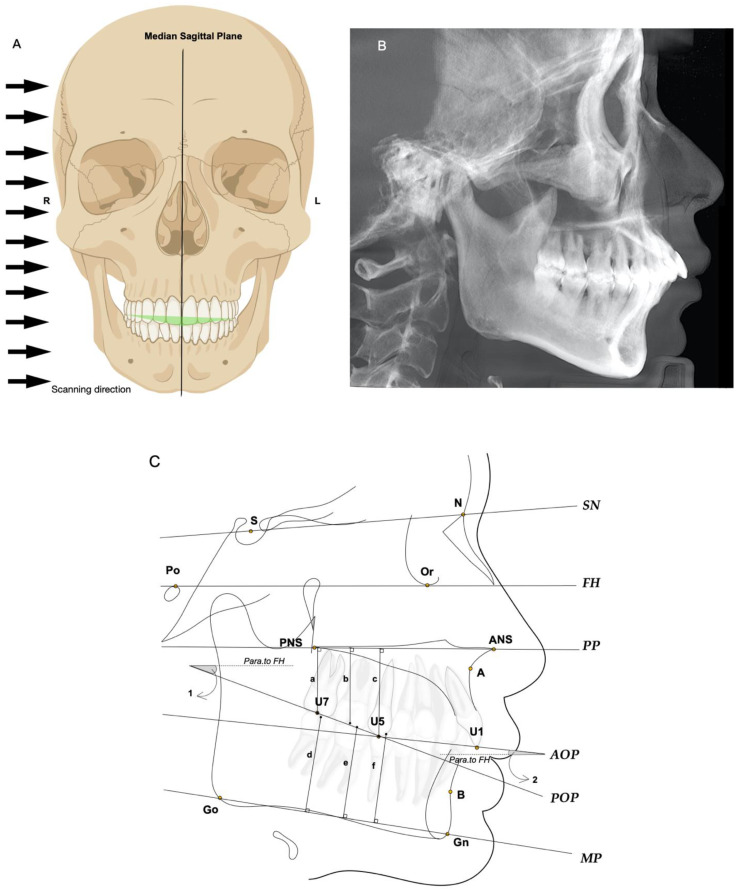
Measurement of the posterior tooth height (U5-PP, U6-PP, U7-PP, L5-MP, L6-MP, L7-MP) and the occlusal plane angle (AOP-FH, POP-FH). (**A**) The method of projecting the 3D reconstruction image into the midsagittal plane from right to left; (**B**) The lateral cephalogram formed after projection; (**C**) Tracing diagram of the lateral cephalogram. It shows the anatomical reference landmarks and measurement planes used for cephalometric measurements. Landmarks are defined as follows: sella point (S); nasion point (N); anatomical porion point (Po); orbitale point (Or); anterior nasal spine point (ANS); posterior nasal spine point (PNS); subspinale point (**A**); supramental point (**B**); gnathion point (Gn); gonion point (Go); the buccal cusp of the maxillary second premolar (U5); the midpoint of the maxillary first molar at the occlusal surface (U6, not shown); the midpoint of the maxillary second molar at the occlusal surface (U7); the buccal cusp of the mandibular second premolar (L5, not shown); the midpoint of the mandibular first molar at the occlusal surface (L6, not shown); the midpoint of the mandibular second molar at the occlusal surface (L7, not shown); the incisal edge point of the maxillary central incisor (U1). In addition, a-f represent U7-PP, U6-PP, U5-PP, L7-MP, L6-MP, and L5-MP, respectively, while angle 1 and angle 2 represent POP-FH and AOP-FH, respectively.

The posterior tooth height (U5-PP, U6-PP, U7-PP, L5-MP, L6-MP, L7-MP) [62] and the occlusal plane angle (AOP-FH, POP-FH) [36] were measured on the lateral cephalogram from CBCT by projecting the 3D reconstruction image into the midsagittal plane from right to left (Figure 4A,B). U5, U6, and U7 represent the buccal cusp of the maxillary second premolar, the midpoint of the maxillary first molar at the occlusal surface, and the midpoint of the maxillary second molar at the occlusal surface, respectively. L5, L6, and L7 represent the points of the mandibular posterior teeth. The palatal plane (PP) was determined using a line drawn from the anterior nasal spine point (ANS) to the posterior nasal spine point (PNS). U5-PP, U6-PP, and U7-PP were defined as the vertical distance from the U5, U6, and U7 points to PP, respectively, while L5-MP, L6-MP, and L7-MP were defined as the vertical distance from the L5, L6, and L7 points to MP, respectively (Figure 4C).

The Frankfort horizontal plane (FH) was determined using a line drawn from the orbitale point (Or) to the anatomical porion point (Po), which was generally parallel to the ground level. The posterior occlusal plane (POP) was determined by the line between U5 and U7, and the anterior occlusal plane (AOP) was determined by the line between U5 and the incisal edge point of the maxillary central incisor (U1). AOP-FH was defined as the angle between FH and AOP while POP-FH was defined as the angle between FH and POP. The location of the anatomical reference landmarks and specific measurement methods can be seen in the schematic diagram of the lateral cephalogram (Figure 4C).

**Figure 5:**
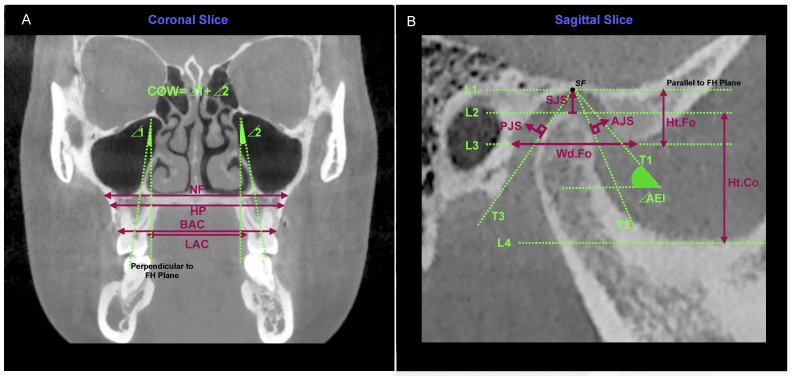
Measurement of the transverse width (NF, HP, BAC, LAC), the curve of Wilson (COW), and TMJ indexes. (**A**) NF: maxillary basal bone width at tangent to the nasal floor; HP: maxillary basal bone width at tangent to the hard palate; BAC: maxillary alveolar bone width between the bilateral buccal alveolar ridge; LAC: Maxillary alveolar bone width between the bilateral lingual alveolar ridge. The curvature of COW was defined as the sum of the bilateral angles between the line connecting the central fovea and root bifurcation and the line perpendicular to the FH plane. (**B**) Seven tangent lines were drawn as mentioned in the text. Ht.Co: vertical distance between L2 and L4; Ht.Fo: vertical distance between L1 and L3; Wd.Fo: horizontal distance between the intersection point as mentioned and the tangent point of L3; AEI: the angle between the T1 and FH of the TMJ index; joint spaces were measured using the Kamelchuk method.

After the measurement above, the head position was re-adjusted in three dimensions, passing through the FH plane in the bilateral sagittal position, and maintaining the head position in the subsequent measurement. Regarding the transverse width (NF, HP, BAC, LAC) [63] and the curve of Wilson (COW), they were all measured on the coronal slice passing through the maximum section of the maxillary first molars (Figure 5A). COW itself is defined as a convex downward curve formed by connecting the buccal and lingual cusps of the homonymous maxillary molars on both sides. In this study, we measured the curvature of COW [64] by drawing two lines connecting the bilateral central fovea and root bifurcation, respectively, and the sum of the included angles between each line and the line perpendicular to FH was defined as the curvature of COW. The curve of Spee (COS) itself is defined as a concave upward curve formed by the incisal edge of the mandibular central incisors passing backwards through the cusp of the mandibular canines, the buccal cusps of the mandibular premolars, and the mesial and distal buccal cusps of the mandibular molars. The measurement of COS was carried out in 3D reconstruction images using 3D points and calibration. Different from the COW measured by the angle, the depth of COS [64] was more clinically meaningful. A plane was determined at the incisal edge of the mandibular central incisors and the distal buccal cusp of the bilateral mandibular second molars (Figure 3A and Figure 4A show this plane in green). The depth of COS was considered as the maximum distance from the lowest point of the bilateral COS to this plane.

#### 2.3.3. Temporomandibular Joint Indexes

There were 7 items in total, and all measurements were completed on the same measurement plane. The measurement plane (condylar sagittal plane) was determined by moving layer by layer in the horizontal and coronal views until the inner and outer diameter of the condyle was the largest in the two views. Then, in the sagittal view, scanning from right to left step by step was performed until the slice passed through the highest point of the glenoid fossa (Figure 5B).

The measurements of the joint indexes [65] were conducted as follows:

1. Draw four parallel lines of FH from the superior to the inferior, tangent to the glenoid fossa, condyle, the lowest end of the articular eminence, and the sigmoid notch of the mandible, respectively, named L1, L2, L3, and L4. The vertical distance between L2 and L4 was defined as the condyle height (Ht.Co) and the vertical distance between L1 and L3 was defined as the fossa height (Ht.Fo). L3 intersected the posterior wall of the fossa, and the horizontal distance between the intersection point and the tangent point was defined as the fossa width (Wd.Fo).
2. Taking the tangent point of L1 (superior fossa (SF) point) as the starting point, draw three new tangent lines from the anterior to the posterior, which were tangent to the posterior slope of the articular eminence, the anterior edge of the condyle, and the posterior edge of the condyle, respectively, named T1, T2, and T3. The angle between T1 and FH was defined as the articular eminence inclination (AEI).
3. Joint spaces were measured using the Kamelchuk method [66]. The distance between the tangent point of L1 and L2 was measured as the superior joint space (SJS). The perpendicular distance from T2 tangent point to the posterior slope of articular eminence was defined as anterior joint space (AJS), and the perpendicular distance from T3 tangent point to the posterior wall of fossa was defined as posterior joint space (PJS).

### 2.4. Statistical Analysis

SPSS 26 software (IBM Corp., Armonk, NY, USA) was applied. All the items were measurement data, expressed as (mean ± standard deviation). Kolmogorov–Smirnov was utilized to examine whether the analysis data were in accordance with a normal distribution. The 10 patients with the short-faced pattern were compared before and after injection. A paired t test was performed for those with a normal distribution and Wilcoxon paired test was performed for those with a non-normal distribution. Meanwhile, the symmetry of the bilateral indexes was also checked using a paired t test. In addition, we also carried out correlation analysis for each measurement index before injection. Pearson correlation analysis was conducted for the measurement items conforming to a normal distribution, and Spearman rank correlation analysis was conducted for those that did not conform to a normal distribution. The bilateral test level was set at α = 0.05 and *p* < 0.05 was considered statistically significant.

## 3. Results

### 3.1. Changes in the CBCT Masticatory Complex

#### 3.1.1. Changes in the Masseter Muscle Thickness

**Figure 6:**
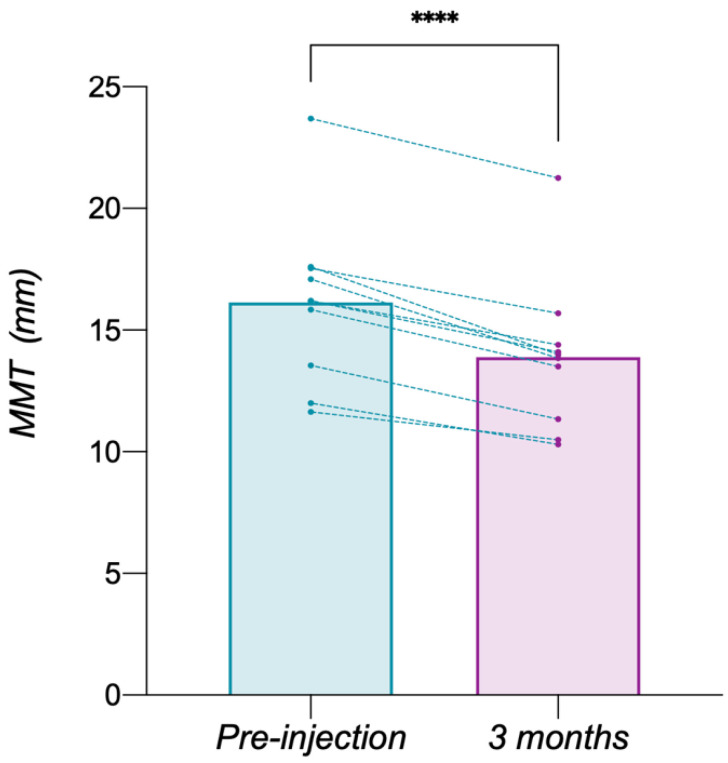
Changes in the masseter muscle thickness. It illustrates a significant reduction in MMT 3 months after BTX-A injection (****: *p* < 0.0001).

There was no significant difference in the bilateral MMT before or after BTX-A injection (*p* > 0.05), indicating symmetrical bilateral masseter muscle in all patients. Therefore, the average value of the bilateral MMT was utilized in this study. The average MMT of 10 patients with low angle before and 3 months after BTX-A injection was 16.14 ± 3.44 mm and 13.90 ± 3.14 mm, respectively, significantly reduced by 2.25 ± 0.73 mm (*p* < 0.0001) (Figure 6).

#### 3.1.2. Changes in Dental Occlusion

**Figure 7:**
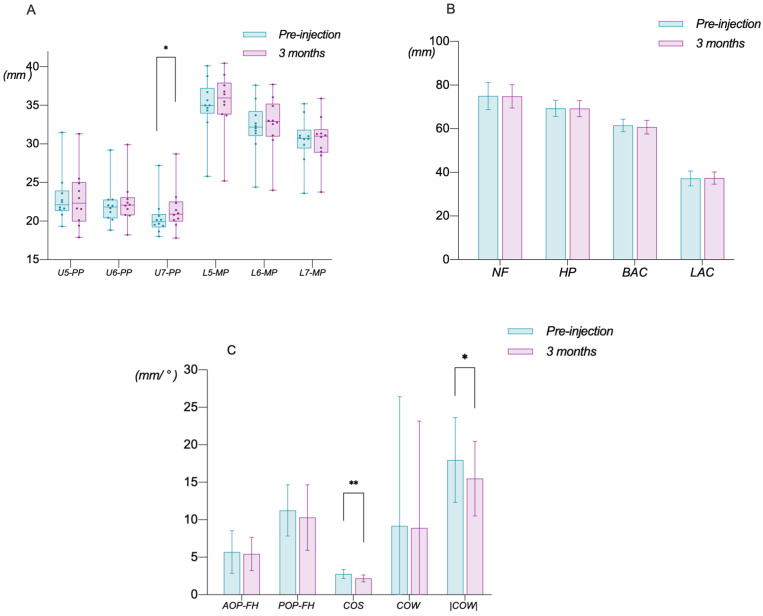
Changes in dental occlusion. (**A**) Variations in the posterior tooth height. It illustrates that U7-PP of 10 patients significantly increased 3 months after BTX-A injection while U5-PP, U6-PP, L5-MP, L6-MP, and L7-MP showed no obvious changes. (**B**) Variations in the transverse width. It illustrates no significant differences in both the basal bone (NF, HP) and alveolar bone (BAC, LAC) before and 3 months after BTX-A injection. (**C**) Variations in the functional occlusion. It illustrates that neither AOP-FH nor POP-FH showed a significant change while the depth of COS showed a significant decrease. As for the curvature of COW, its value itself does not change significantly, but a significant flattening was found when the change in the absolute value of COW before and 3 months after BTX-A injection was compared (*: *p* < 0.05; **: *p* < 0.01).

In terms of the variation in the posterior tooth height, U7-PP increased from 20.51 ± 2.56 mm before BTX-A injection to 21.52 ± 2.92 mm after injection, with a significant difference of 1.01 ± 1.27 mm (*p* < 0.05) (Figure 7A). These results suggested that BTX-A caused compensatory elongation of the maxillary second molars by about 1mm, and elongation of the maxillary posterior teeth far from the masseter muscle fibers and all mandibular posterior teeth was not evident, as U5-PP, U6-PP, L5-MP, L6-MP, and L7-MP showed no significant change. There were also no significant differences in NF, HP, BAC, and LAC before and 3 months after Botox injection (Figure 7B), indicating the unavailability of BTX-A in the reconstruction of both the maxillary basal bone width and maxillary alveolar bone width.

Neither AOP-FH nor POP-FH showed significant changes, despite a nearly 1° flattened value of the POP. However, the changes in the occlusal curve were striking, as the depth of COS decreased from 2.76 ± 0.60 mm to 2.17 ± 0.46 mm after BTX-A injection, significantly decreasing by 0.59 ± 0.55 mm (*p* < 0.01), suggesting an astonishing leveling effect of COS. As for the curvature of COW, its value itself did not change significantly. However, considering the existence of a negative value, when comparing the changes in the absolute value of COW before and after injection, we also found a significant flattening (*p* < 0.05), decreasing from 17.97 ± 5.66 ° to 15.49 ± 4.97 °. This indicates the occurrence of an upright effect of 2.48 ± 2.87 ° in the inclination angle of the maxillary posterior teeth (Figure 7C).

#### 3.1.3. Changes in TMJ

**Figure 8:**
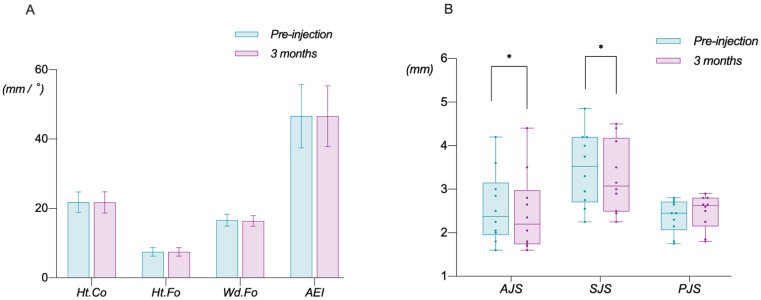
Changes in TMJ. (**A**) Variations in the joint structure. It illustrates no significant changes in Ht.Co, Ht.Fo, Wd.Fo, or AEI 3 months after BTX-A injection. (**B**) Variations in the joint space. It illustrates that AJS and SJS were significantly reduced 3 months after BTX-A injection while PJS showed an insignificant increase (*: *p* < 0.05).

The TMJ underwent the same bilateral measurement as that of the masseter muscle. As each index of the bilateral joint exhibited no significant difference before and after injection (*p* > 0.05), the average value was adopted. In terms of the joint structure, there were no significant changes in Ht.Co, Ht.Fo, Wd.Fo, or AEI after BTX-A injection in 10 patients with a short-faced pattern (Figure 8A), indicating that BTX-A injection in this study did not promote remodeling of the TMJ structure, regardless of the condylar height, the fossa height and width, and the posterior slope of the articular eminence. In terms of the joint space, AJS and SJS were significantly reduced (*p* < 0.05) by 0.13 ± 0.16 mm and 0.21 ± 0.24 mm, respectively, suggesting a conceivable anterior-upper rotational shift of the condyle position, while PJS showed an insignificant increase of about 0.10 mm (Figure 8B).

### 3.2. Correlation Analysis of the Components of the Masticatory Complex in Short-Faced Patients

**Figure 9:**
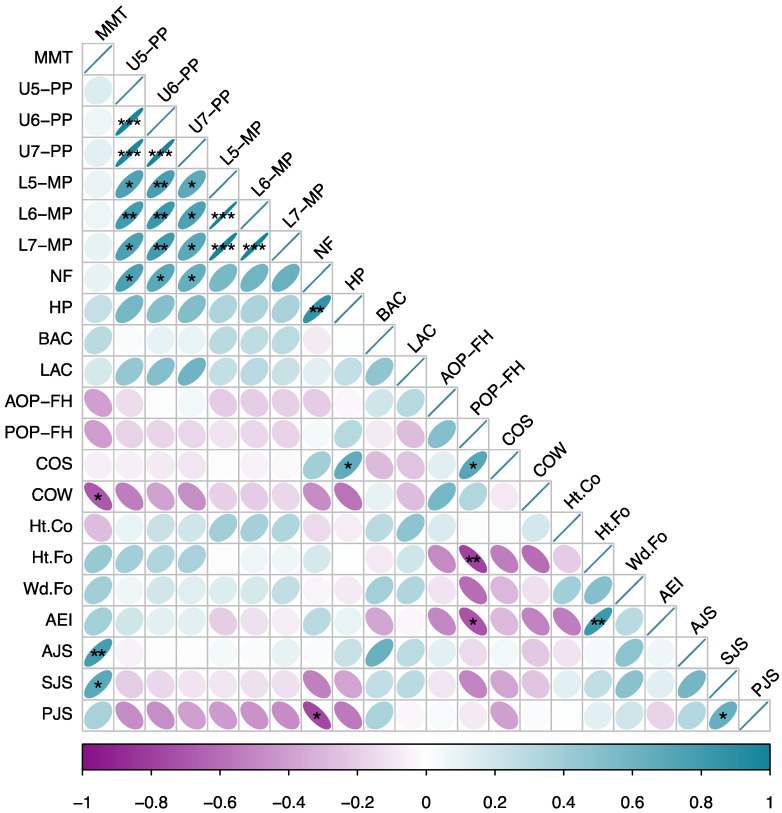
Correlation heatmap of the masticatory complex. Positive correlation is represented by cyan ellipses, whereas negative correlation is represented by purple ellipses, with a deeper hue indicating a stronger correlation. Specifically, the darker the cyan, the closer the r is to 1, and the darker the purple, the closer the r is to −1. Similarly, when the correlation varies, the size of the ellipse changes. The closer the r is to 1 (cyan) or −1 (purple), the closer the ellipse is to a line, whereas the closer the r is to 0, the closer the ellipse is to a perfect circle. Correlations with significant differences are marked in the figure. (*: *p* < 0.05; **: *p* < 0.01; ***: *p* < 0.001).

Pairwise correlation analysis was conducted between all variables before BTX-A injection, and a correlation heatmap was generated (Figure 9). We found that MMT exhibited a significantly negative correlation with COW (*p* < 0.05) while a significantly positive correlation with AJS and SJS (*p* < 0.01, *p* < 0.05). After synthetically considering the absolute value of the correlation coefficient *r* and the clinical significance, COW was taken as the occlusal index and AJS as the TMJ index in the final regression equation.

Eventually, we performed multiple linear regression analysis with pre-injection MMT as the dependent variable while COW and AJS as independent variables, and obtained the regression model: MMT = 10.08 − 0.11 COW + 2.73 AJS. Here, all variables and constants possessed robust statistical significance in the regression equation (*p* < 0.01), and the whole regression model exhibited extremely high significance (*p* = 0.0003). The high value of the adjusted R^2^ indicated the capability of the regression model to explain the variation of MMT of about 87.1% (Table 1).

## 4. Discussion

### 4.1. Effects of BTX-A on Homeostasis Reconstruction of the Masticatory Complex

#### 4.1.1. BTX-A and Masticatory Muscle

Patients with a short-faced pattern generally experience the problem of benign masseter hypertrophy due to the mutual shaping of the muscle and bone in the process of growth and development [21,22,25]. BTX-A is regarded as the most effective approach to treat benign masseter hypertrophy [41,67]. In this study, BTX-A significantly promoted a reduction in the thickness of the masseter muscle in 10 patients, consistent with the previous results obtained from B-ultrasound assessment by Diracoglu et al. [68], CT assessment by Hong et al. [39], and 3D laser scanning assessment by Lee et al. [69]. The results above demonstrate the effectiveness of BTX-A in masticatory muscle adjustment, which also provide the premise for subsequent study, and verify the effective diagnostic value of CBCT for the masseter muscle [60].

Zhang et al. [70] injected BTX-A into the masseter muscle of patients with bruxism and found that the biting force in the maximum intercuspal position significantly decreased, which directly proved the efficiency of the BTX-A treatment in reducing the biting force. A similar property was indirectly verified through electromyography by Lee et al. [71]. Through 3D CT reconstruction, Hong et al. [39] detected changes in the cortical bone thickness at the insertion sites of the masseter muscle and temporalis muscle after BTX-A injection. The imaging evidence also indirectly reflected a reduction in the muscle functional load. Although the inhibitory effect of BTX-A on neuromuscular junction transmission is not permanent, with a period of about 3 months from the peripheral nerve germination to functional recovery of the neuromuscular complex, it has also been reported that repeated injections of BTX-A help maintain reduced biting force [72].

#### 4.1.2. BTX-A and Teeth and Occlusion

In untreated patients, the functional contraction direction of the masseter muscle should be at a stable angle to the functional occlusal plane of the posterior teeth, generating three-dimensional forces on the occlusal surface of the posterior teeth, which are composed of vertical, anterior, and lateral force [73]. Occlusal treatment (such as orthodontic treatment) is a dynamic process in which the original muscle-tooth balance is constantly destructed, and the new balance is rebuilt as the tooth position is modulated. For skeletal high angle patients with a long face, their weak masticatory muscles and low strength provide conditions for an efficient and smooth treatment process as generally soft and light force can counter the old balance [21,37]. On the contrary, for low angle patients with a short-faced pattern, due to the strong masticatory muscles and robust biting forces in the three-dimensional direction, the processes involved during treatment against the vertical force to open bite [29,30], against the anterior force to prevent molar mesial drift and COS deepening [74,75], or against the lateral force to control the maxillary width and buccolingual molar inclination (COW) are significantly difficult [76,77]. Even if these are achieved, high rates of recurrence and periodontal risks were reported. Therefore, during the early stage of occlusal treatment, the muscle strength and biting force should be adjusted as soon as possible by injecting BTX-A, which can directly affect the load of the occlusal surface of teeth, and then manipulate the changes in the three-dimensional dental eruption state to adapt to the remodeling of balance in a more efficient and healthy way [29].

Most studies on tooth changes caused by BTX injection have focused on animal experiments [49,50,51,52,53]. Multiple previous studies have proved that BTX injection can induce a double decline in the shape and function of masticatory muscles, and that the reduced biting force will lead to manipulation of the tooth height. Tsai et al. [51] injected BTX unilaterally into rat masseter muscle, showing an excessive eruption of the upper and lower molars, in addition to a decrease in the size and weight of the masseter muscle. Choi et al. [49] injected BTX into the unilateral masseter muscle and temporal muscle of developing rats to weaken the muscle force, and discovered that compensatory alveolar eruption caused deviation in the occlusal plane. Navarrete et al. [53] found that BTX injection administered to rabbits elicited excessive tooth eruption, and argued that it would affect facial morphology changes, such as increasing the vertical dimension or tendency of a long-faced pattern. Considering the differences in the anatomy of the muscles, jaw, and drug metabolism between humans and different animals, this evidence is unsuitable for direct extrapolation to humans but confirms the feasibility of injecting BTX into the masticatory muscles to induce paralysis as a means of adjusting occlusion. Currently, in clinical studies, only some examples of BTX-A in maintaining occlusal stability after orthognathic surgery have been reported [78]; the efficacy of BTX-A in non-surgical occlusal therapy has not been studied. Dai et al. [79] found that the biting force was almost distributed on the molars during static masticatory movement, in which the force on the incisors was almost zero. Therefore, this study mainly focused on the posterior teeth. The U7-PP of 10 patients significantly increased 3 months after BTX-A injection, indicating compensatory elongation of the maxillary second molar while the height of other posterior teeth showed no obvious changes. This may indicate that the lower bone mineral density of the maxilla, and the more abundant nerves and vessels in the maxilla make it more vulnerable to adjustment and remodeling by external forces than the mandible. In addition, the physical distance between the masseter fibers and teeth will also directly affect the eruption state of the teeth. In terms of the transverse width, NF, HP, BAC, and LAC exhibited no significant changes before and after injection, indicating that the basal and alveolar bone widths of adult patients were relatively stable and unaffected by changes in the masseter muscle strength. This seems to be inconsistent with Kiliaridis et al.’s study [80], possibly because the latter samples were involved in the juvenile development period and gender differences were differentiated.

In addition to static occlusal indexes, modern concepts of functional occlusion pay more attention to balance and stability during dynamic mastication. The effect of BTX-A on functional occlusion in animals or humans remains to be studied, and this study is an innovative exploration. In normal chewing and grinding movement, the mandible moves laterally or forward, the posterior teeth should be separated, and the anterior teeth contact to form the canine guide (lateral movement) or incisor guide (forward movement), where the anterior teeth can protect the posterior teeth during functional movements [10,81]. However, short-faced people often have a steep posterior occlusal plane or deep occlusal curves, meaning a high possibility of occlusal interference [35], which may cause tooth abrasion, periodontal disease, and TMD. In this study, the process of dynamic occlusion was reflected by two occlusal planes and two occlusion curves. Under extremely ideal conditions, COS and COW should be represented as an arc located on the same sphere (Monson sphere) [64]. Here, both COS and COW were significantly flattened 3 months after Botox injection. Apart from the influence of the orthodontic force itself, the weakened occlusal force broke the original muscle-tooth balance and promoted the three-dimensional movement of teeth in a more efficient way. Studies [82] have demonstrated the close relation between the occlusal curve and masticatory function. Generally, individuals who have flatter curves may obtain a better food mixing capability, higher masticatory efficiency, less occlusal interference, and lower incidence of periodontal disease and TMD. As for the occlusal planes, neither POP nor BOP showed significant changes, which may be related to the fact that the interference of the sagittal skeletal type was not excluded in this study, with a variety of sagittal skeletal types. Professor Sato [36] believed that the increased POP-FH was the source of the occurrence of the Class II pattern; the crux for successful treatment in such patients was to flatten the POP [83]. It has been found that POP does tend to flatten after BTX-A injection, which may be related to the orthodontic force itself or the compensatory elongation of the maxillary second molars caused by masseter paralysis. The variation characters in the occlusal curves and occlusal planes reflect the dynamic reconstruction process of the stomatognathic system, and BTX-A injection may effectively accelerate the rate of homeostasis reconstruction in low angle patients with a lower incidence of complications [32].

#### 4.1.3. BTX-A and TMJ

According to the international classification of DC/TMD, TMD can be divided into two categories [84,85]. The first category is pain diseases, which generally involve joint pain itself and pain secondary to masticatory muscle hyperfunction [86,87,88]. BTX-A has a good therapeutic effect on pain diseases as it can inhibit the release of local pain neuropeptides, such as substance P, calcitonin gene-related peptide, glutamate, and transient receptor potential V1, which has also attracted multiple clinical studies [86,87,88]. Due to the positive therapeutic effect of this kind of disease, this study did not continue its exploration.

The second type is joint organic disease. TMJ remodeling often occurs after mechanical load changes in the stomatognathic system, mainly including the process of morphological adaptation (such as changes in the condyle bone) and spatial adaptation (such as changes in the position of the condyle and joint disc) [89]. The former is often applied as a model of paralysis in animal experiments, where BTX-A induces bone changes over a short period of time. Rafferty et al. [90] found bone loss of the mandibular condyle in rabbits after an injection of BTX-A into the unilateral masseter muscle, which was also found in the masseter muscle attachment area in rats by Tsai et al. [52] and Kun-Darbois et al. [91]. However, due to the discrepancy between animal and human bone biomechanics, the effect of BTX-A injection on the condylar bone in the human body remains controversial. Based on 3D CT reconstruction, Hong et al. [39] found that reduced muscle functional load affected the cortical bone mass of the condyle, which was more significant in postmenopausal women. While Raphael et al. [92] seemed to reach the opposite result, finding no clinically significant changes in the density or volume of the TMJ complex in women with TMD who had received at least two (and often three) cycles of BTX-A treatment in the past year. Since the experiments above were mostly based on TMD patients who had repeated injections of botulinum, this study aimed to explore whether a single botulinum toxin injection would adversely affect the condylar bone in the normal population, which may run counter to our original intention. Fortunately, no bone changes in TMJ were found in the 10 patients 3 months after the single BTX-A injection, which is consistent with the findings of Lee et al. [55], regardless of whether the condyle, glenoid fossa, or articular eminence was examined. During the follow-up of the 10 patients, no adverse indications such as TMJ clicks and pain were reported. On the contrary, two patients said that their mild bruxism symptoms were relieved, with acid swelling of the masticatory muscles in the morning disappearing, which actually resulted from the weakened biting force caused by masseter muscle atrophy. The weakened biting force reduced the load on the TMJ area, which was conducive to the decompressed and balanced repair environment of the TMJ.

Moreover, this experiment innovatively involved the joint space indexes to verify the dual response of TMJ to the mechanical load. In addition to morphology adaptation, there is also spatial relocation [23]. The masticatory muscle function is well known to be critical for proper TMJ development during ontogeny [93]. In patients with skeletal low angle, the joint structure is usually thick and broad due to strong muscle force while the condyle position is often posterior and inferior due to excessive counterclockwise closure of the mandible. An abnormal condyle position and abnormal occlusal relationship are risk factors for TMD [94]. The 10 patients in this study showed a reduction in AJS and SJS after BTX-A injection, which is conducive to the stabilization of the condyle in the central relation (CR) position. As the father of modern occlusions, Dr. Roth [94] believed that a stable stomatognathic system should be equipped with stable joint position and coordinated occlusal relationship, as Dr. Dawson summed up that when the upper and lower teeth are in the maximum intercuspal position, the condyle should be at the front and top of the glenoid fossa [3]. Therefore, BTX-A can coordinate the condyle position with the final target position of the occlusal treatment, contributing to the long-term stability of the curative effect and low rates of TMD.

### 4.2. Preliminary Study on the Homeostasis Mechanisms of the Masticatory Complex

To explore the homeostasis mechanisms of the masticatory complex in short-faced patients, we analyzed the pairwise correlation between all variables before injection. The MMT was defined as a novel entry point for the homeostasis balance of the occlusal treatment to explore how the changes in occlusion and TMJ under the state of physiological balance manipulate and adapt to the changes in the masticatory muscle.

When selecting variables for regression analysis, not only the absolute value of correlation coefficient r should be considered but also the clinical factors and possible collinearity problems. For example, in addition to COW’s robust correlation, there also exists a certain correlation between COW and COS depending on the clinical conditions, and the correlation heatmap showed a significant positive correlation between COS and POP-FH (*p* < 0.05). Therefore, the final inclusion of COW was consistent with both the experimental results and the clinical significance. As for why AJS instead of SJS was chosen, in addition to the greater r of AJS, we also considered the correlation of SJS and PJS depicted in the heatmap (*p* < 0.05). Considering the correlation of PJS and AJS in the clinic, AJS and SJS may show collinearity in a clinical sense, so eventually only AJS was included as the only TMJ index. Consequently, the whole regression model was highly significant (*p* = 0.0003) and had an explanation capability of 87.1% of the clinical outcomes.

Here, the homeostasis of the masticatory complex in patients with a short-faced pattern was intuitively and quantitatively described firstly by means of a multiple linear regression model. As mentioned above, mastication is one of the main oral functions, and its efficient performance requires a healthy and stable stomatognathic system. It generally consists of occlusion, TMJ, and masticatory muscles, which are defined as a functional complex in this study. They are interrelated and have mutual influence, forming a stable balance triangle to ensure the normal operation of the stomatognathic system under normal physiological conditions, where change in any one of the links will lead to an imbalance of the triangular relationship. When the complex exceeds the adaptability of the body, corresponding diseases will arise from the weakest link [10,17,19], such as malocclusion, TMD, myofascial pain, etc. These diseases are collectively referred to as occlusal diseases, the treatment of which is so-called occlusal treatment, where a holistic treatment concept is apparently required.

### 4.3. Limitations

Due to the innovative significance of this study, as a brand-new exploration of BTX-A in occlusal therapy, the sample size included was limited. Moreover, similar to the new application of BTX in other clinical fields, it lacked the evidence support of a long-term follow-up, such as the injection dose, injection frequency, and side effects.

### 4.4. Future Perspective

More research, particularly well-designed randomized controlled trials with high evidence levels, is needed in the future to verify the reliability and long-term stability of the above findings by expanding the sample size, lengthening the follow-up time, and increasing the number of BTX-A injections. Simultaneously, an individualized BTX-A dosage, injection method, administration frequency, and cycle should be researched further in order to provide a novel adjuvant treatment approach for short-faced individuals within a safe range of occlusal therapy.

## 5. Conclusions

BTX-A can provide a more favorable muscular environment for short-faced patients through adjustment of the biting force, contributing to the reconstruction of healthier homeostasis of the masticatory complex. However, further research is required to validate these findings.

## Figures and Tables

**Table 1:** Regression analysis of MMT.

	Standardization Coefficient β	Significance	Adjusted R^2^	*p* Value
(Intercept)	10.08	0.0002	0.871	0.0003
COW	−0.11	0.003		
AJS	2.73	0.001		

MMT = 10.08 − 0.11 COW + 2.73 AJS.

## Data Availability

The data presented in this study are available on request from the corresponding author due to restrictions e.g. privacy or ethical.

## Abbreviations

BTX-A	botulinum toxin-A	FH	frankfort horizontal plane
MS	masticatory system	AOP	anterior occlusal plane
SS	stomatognathic system	POP	posterior occlusal plane
TMJ	temporomandibular joint	COS	curve of Spee
TMD	temporomandibular joint disorders	COW	curve of Wilson
CBCT	cone-beam computed tomography	AEI	articular eminence inclination
MMT	masseter muscle thickness	AJS	anterior joint space
MP	mandibular plane	SJS	superior joint space
PP	palatal plane	PJS	posterior joint space
